# Psychotherapy training in psychiatry: a systematic review on the early career psychiatrists’ interests and opportunities

**DOI:** 10.1192/j.eurpsy.2025.10044

**Published:** 2025-06-16

**Authors:** Selin Tanyeri Kayahan, Mustafa Dinçer, Jesper Nørgaard Kjær, Thomas Gargot

**Affiliations:** 1 Psychiatry Clinic, Republic of Turkey Ministry of Health Yalvaç Public Hospital, Isparta, Turkey; 2 Department of Child and Adolescent Psychiatry, Aydın Adnan Menderes University Medical Faculty, Aydın, Turkey; 3 Psychosis Research Unit, Aarhus University Hospital, Aarhus, Denmark; 4 Department of Clinical Medicine, Aarhus University, Aarhus, Denmark; 5 Department of Child and Adolescent Psychiatry, EXcellence Center in Autism and Neurodevelopmental Disorders, Tours ExAC-T, Tours, France; 6 Tours University, INSERM, Imaging Brain & Neuropsychiatry iBraiN U1253, 37032, Tours, France

**Keywords:** early career psychiatrist, psychiatry resident, psychiatry training, psychotherapy, trainee

## Abstract

**Background:**

Psychotherapy plays a crucial role in mental healthcare. Integrating evidence-based practices into treatment guidelines highlights the need for basic psychotherapy competence in psychiatry training. While programs set minimum requirements for psychotherapy training in line with the recommendations of the World Psychiatric Association or accreditation bodies like the European Union of Medical Specialists, implementation is often inconsistent, and resources are limited. This systematic review explores early career psychiatrists’ (ECPs) views, interests, and available opportunities for psychotherapy training worldwide.

**Methods:**

We systematically searched MEDLINE, Scopus, and PubPsych for survey-based studies on ECPs’ perspectives on psychotherapy training, following the Preferred Reporting Items for Systematic Reviews and Meta-Analyses guidelines. Of 31,281 studies screened, 48 articles were included. Quality assessment was conducted using the Quality Assessment Checklist for Survey Studies on Psychology, and the findings were summarized through narrative synthesis.

**Results:**

Included studies were from Europe (24, 50%), United States (12, 25%), Western Pacific (6, 12.5%), South-East Asia (4, 8.3%), Eastern Mediterranean (1, 2%), and Africa (1, 2%), with a total of 7,196 participants. Thirty-one studies on ECPs’ interest in psychotherapy training found that 57–80% were interested in psychotherapy, 67–92% viewed being a psychotherapist as part of their psychiatrist identity, and 88–97.7% supported its inclusion in psychiatry training. Training opportunities varied by country and institution, with cognitive behavioral therapy and psychodynamic psychotherapy being primary modalities.

**Conclusion:**

Improving psychiatrists’ access to evidence-based, culturally adapted psychotherapy training is essential. Educational activities offered by training institutions and professional organizations can play a key role in supporting ongoing professional development.

## Introduction

Psychotherapy is a key component of comprehensive mental health care [1], proven effective in treating disorders like depression [2] and anxiety disorders [3], as well as helping individuals cope with loss, trauma, and life challenges. With the integration of evidence-based psychotherapy practices into international treatment guidelines [4, 5], training in psychotherapy is essential for psychiatrists to expand their therapeutic skills and lead mental healthcare teams [6]. As a result, most psychiatry training programs set minimum requirements for trainees to develop core competencies in psychotherapy.

Psychotherapy skills are generally categorized into three levels: generic, basic practitioner, and advanced. At the generic level, psychiatrists should form a therapeutic alliance and evaluate their emotional reactions with a nonjudgmental attitude. Basic training equips psychiatrists to deliver supervised psychotherapy in one modality and gain knowledge of other major approaches. Advanced training is designed for those with a strong interest in psychotherapy, helping to identify and nurture potential candidates [7].

The World Psychiatric Association (WPA) recommends that training in basic psychotherapy knowledge in the first year, advanced psychotherapy knowledge in the second year, and gaining the skill to be able to deliver effective medium- to long-term psychotherapy during psychiatry training as core competencies [8]. In Europe, the European Union of Medical Specialists (UEMS) Board of Psychiatry considers supervised psychotherapy experience a key competency, defining the first level as “learning in psychotherapy” where psychiatrists gain skills to provide psychologically informed mental health services. Psychiatrists should be resilient practitioners, expert communicators, and motivated therapists, building trust with patients and directing them to appropriate psychological treatments [9]. In the United States, the Accreditation Council for Graduate Medical Education (ACGME) requires competence in supportive, psychodynamic, and cognitive-behavioral psychotherapies (CBT) [10]. However, despite well-defined guidelines, research showed that psychotherapy training remains challenging in psychiatric education [6]. The WPA reported that nearly half of the member countries did not require psychotherapy training [11]. Meanwhile, training hours were decreasing, with limited resources and poor implementation of UEMS recommendations across Europe [12].

In the past decade, accreditation bodies like UEMS have promoted learner- and outcome-focused psychotherapy education and training. Studies on psychiatric trainees’ and early career psychiatrists’ (ECPs) perspectives mainly highlight the opportunities and challenges in the current training models, as well as barriers to accessing psychotherapy training. Key barriers include variability and a lack of standardization in training opportunities, and issues with time, funding, and psychotherapy supervision [12–14].

### Objectives

We aimed to evaluate psychiatric trainees’ and ECPs’ interests, views, and available opportunities for psychotherapy training. Given its importance, we hypothesized that psychiatric trainees and ECPs would demonstrate a high interest in psychotherapy despite the variability and a lack of standardization in training. Our review focused on two key questions:

1. Were psychiatric trainees and ECPs interested in psychotherapy training?
2. What were the rates of psychotherapy training among psychiatric trainees and ECPs, and which types of psychotherapy were most commonly taught?

## Methods

The systematic review protocol was registered with the International Prospective Register for Systematic Reviews (PROSPERO) on August 4, 2024 (registration number CRD42024566882) and reported following the Preferred Reporting Items for Systematic Reviews and Meta-Analyses (PRISMA) guidelines [15].

### Search strategy

Articles were identified through MEDLINE, Scopus, and PubPsych searches with no date or language restrictions. A manual search of reference lists and gray literature further supplemented the database search. Two main search concepts were psychotherapy training and ECP/psychiatric trainee.

### Search terms

The following Boolean search terms were used: (((psychiatrist*) OR (psychiatry trainee*) OR (psychiatric trainee*) OR (psychiatry resident*) OR (early career psychiatrist*)) AND ((psychotherapy training) OR (psychiatry training) OR (psychotherapy) OR (psychotherapy practice) OR (psychotherapy education) OR (psychotherapy residency) OR (psychotherapy competency) OR (talking therapy) OR (supervision))).

### Eligibility criteria

The inclusion criteria were survey-based studies reporting ECPs’ or psychiatric trainees’ perspectives on psychotherapy training, published in peer-reviewed journals. Studies had to meet methodological and content criteria, with reviews, review protocols, and purely theoretical articles excluded. Only studies in English, French, or Turkish were included. To maintain relevance, we focused on studies published after 2000. Articles on specific psychotherapy modalities or techniques and surveys solely about training directors or supervisors were excluded. Studies were eligible if they included psychiatrists among the surveyed professionals, even if other categories of professionals were also involved.

### Study selection and data extraction

**Figure 1.**
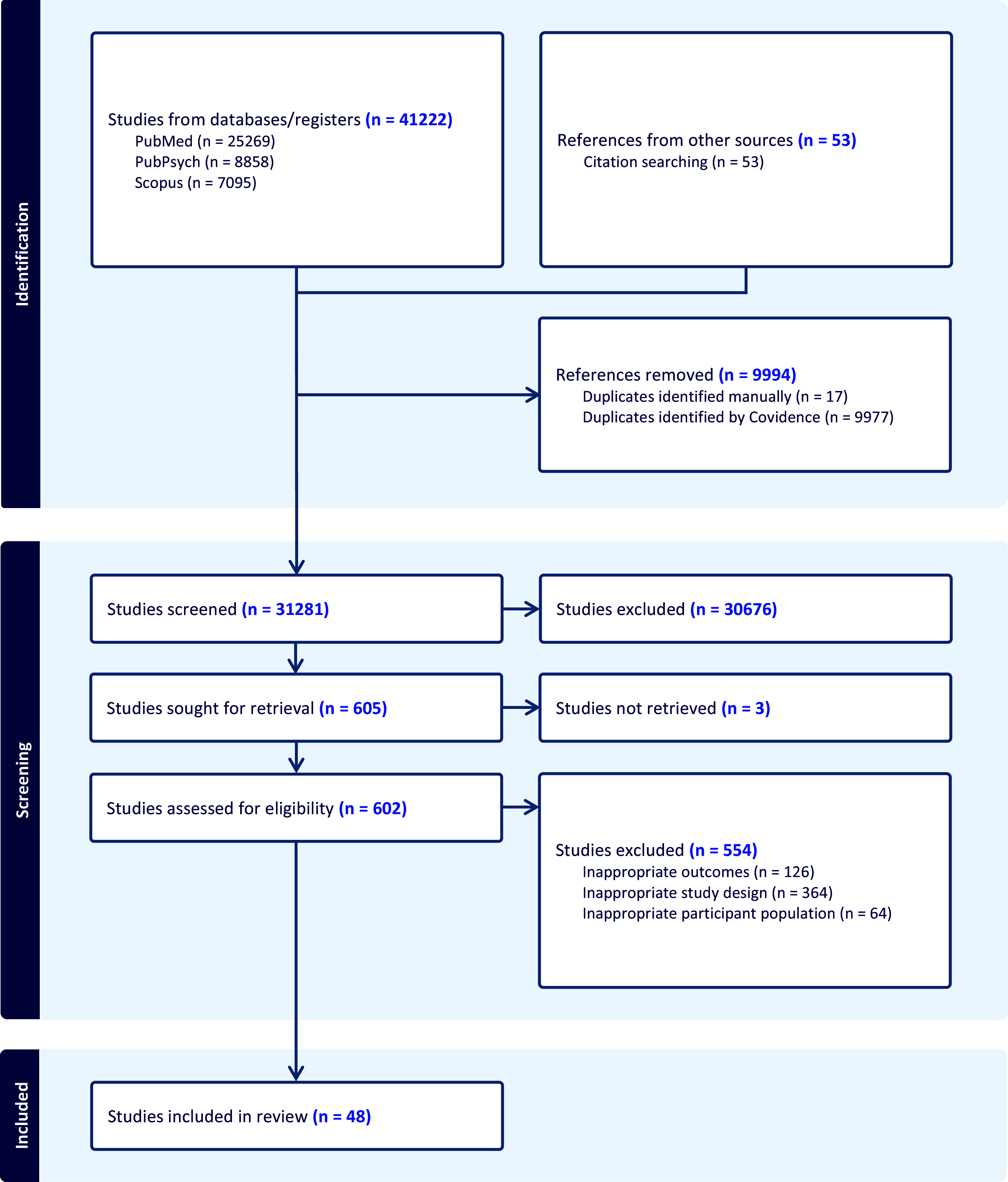
PRISMA flow diagram.

The screening process began with a title and abstract assessment using the inclusion and exclusion criteria via the Covidence tool [16]. The primary reviewer (STK) screened all titles and abstracts, identifying potentially relevant articles. Full-text copies of these articles were retrieved and assessed for inclusion, with further exclusion applied as needed. Additional articles were found through citation searching and a gray literature review. Any discrepancies were resolved through consensus among coauthors. Two authors (STK and MD) were involved in the data abstraction process. The PRISMA flow diagram (Figure 1) illustrates the search protocol, screening, eligibility, and final selection process.

### Data synthesis

Due to the heterogeneity of the included studies in terms of methods, participants, and survey instruments, a narrative synthesis approach was used. The evidence was summarized and categorized by data origin, participants, key findings, and recommendations in a literature table organized by World Health Organization (WHO)-defined regions [17]. The study results were analyzed and interpreted based on the review’s initial research questions. A comparison between high-income countries (HICs) and low- and middle-income countries (LMICs) was made regarding the main findings.

### Quality assessment

The quality of included studies was assessed using the Quality Assessment Checklist for Survey Studies in Psychology (Q-SSP), with studies receiving an acceptable quality score if they met at least 70% of the applicable criteria [18]. Two reviewers (STK and MD) independently applied Q-SSP, modifying items as needed for study design (see Supplementary Materials). For qualitative studies, the Evaluation Tool for Qualitative Studies was used [19].

## Results

The previously outlined search strategy identified a total of 41,222 articles. After removing 9,994 duplicates, the titles and abstracts of 31,281 studies were screened, and 30,676 were excluded because of irrelevance. Six hundred and five potentially relevant full-text articles were sought for retrieval, among which three were unable to be retrieved. Six hundred and two articles underwent further screening, and 554 were excluded for the following reasons: inappropriate outcomes (*n* = 126), inappropriate study design (*n* = 364), or inappropriate participant population (*n* = 64). Forty-eight articles fulfilled all requirements and were included in this systematic review (Figure 1). Table 1 summarizes the key findings of each included study.

**Table 1.** Psychotherapy training interest and opportunities literature summary

Study	Origin	Population	Interest in psychotherapy	General framework and rates of PT	Types of psychotherapy included in PT
African region					
Adiukwu et al. [20]	Nigeria	Psychiatric trainees (*n* = 79) ECPs (*n* = 29) ***n* = 108** (RR: 42.9%)	–79.6% were aware of the inclusion of PT during psychiatry training	–Psychiatry curriculum lacked structure –71.4% reported PT as mandatory –52.8% had PT, mostly partially –PT was mostly available in psychiatry training, but completion rates were very low (2.8%) –PT was theoretical (90.6%) and self-funded (56.5%) –Duration of PT was <100 h (72.4%)	–CBT was the most taught (90.4%) –Followed by family therapy and psychodynamic psychotherapy
Eastern Mediterranean region					
Eissazade et al. [13]	Iran	Psychiatric trainees (*n* = 66) ECPs (*n* = 46) ***n* = 112** (RR: 28%)	–57.2% thought that PT should be mandatory	–Psychiatry curriculum included 9 months of full-time PT –98.2% PT was included in psychiatry training, mostly mandatory (90.8%), both theoretical and practical (49.1%), and with supervision (82.1%)	–Participants were trained in CBT (57.1%) and psychodynamic psychotherapy –CBT (87.5%), psychodynamic (67.7%), family therapy (20.5%), and interpersonal psychotherapy (20.5%) were the most available modalities
European region					
Margariti et al. [21]	Greece	Psychiatric trainees ***n* = 60** (RR: 71%)	–A considerable number showed a favorable attitude toward psychoanalytic psychotherapy	-	–Psychoanalytic psychotherapy was offered in 90.9% of training centers
McCrindle et al. [22]	UK South West region	Senior psychiatric trainees ***n* = 12** (RR: 100%)	-	–83% of schemes offered PT –One in 12 had PT achieving the standards set out in the relevant PT guidelines –41.6% included theoretical training (6–33 h) –75% included practical clinical training (10–120 h)	–Largely varied across training centers –Individual psychodynamic psychotherapy (66.6%), CBT (50%), family therapy (41.6%), group therapy (8.3%)
O’Mahony and Corvin [23]	Ireland	Psychiatric trainees ***n* = 153** (RR: 61.7%)	–75.8% thought that PT was important in their overall training	–39.2% had formal PT	-
Podlejska-Eyres and Stern [24]	UK East London	ECPs ***n* = 22** (RR: 95%)	–55% reported having a relevant current job regarding their PT –54% wished for greater exposure to psychotherapy during their basic training	–45% had an introductory session on psychotherapy during training in psychiatry –91% had access to regular theoretical seminars, 95% attended –86% had access to case presentation psychotherapy seminars, 89% attended –50% participated in other psychotherapeutically oriented courses outside the training scheme –82% had a psychotherapy job on their rotation	–Long-term psychotherapy (95%), CBT (73%), family/marital therapy (59%), group therapy (45%), cognitive-analytical therapy (41%), brief focal psychotherapy (32%)
Carley and Mitchison [25]	UK Northern region	Psychiatric trainees ***n* = 25** (RR: 61%)	–76% were aware of relevant PT guidelines	–88% received satisfactory theoretical teaching in psychodynamic psychotherapy and CBT –52% reported receiving additional PT courses –96% received practical training in interview skills	–48% received theoretical education in family therapy, 44% in group therapy, 8% in interpersonal psychotherapy –76% received training in psychodynamic formulation and 52% in group, couple, or systemic therapy
Pretorius and Goldbeck [26]	UK Scotland	Psychiatric specialist registrars ***n* = 78** (RR: 66%)	–80% were interested in further psychotherapy training, 65% desired to develop a special interest in psychotherapy, with cognitive therapies being the most popular	–33% met relevant PT requirements –49% had competence in at least one modality of psychotherapy	–10% had competence in transference-based therapies, 26% in cognitive therapies, 13% in group or family therapies –9% were qualified in cognitive therapies, 8% in integrative, and 5% in group or family therapies
Lotz-Rambaldi et al. [27]	Europe	Chief of training and the representatives of trainees from 22 countries ***n* = 424**	-	–95% of the countries offered structured theoretical PT in national training schemes –PT was 86% a mandatory part of the training program, other than in Turkey (35%), and partly in Austria (56%) and in Spain (60%) –58% of the centers reported that theoretical PT for at least 120 h was mandatory –97% provided structured theoretical PT –Practical PT in nearly all centers included contact with individuals (97%), with families (86%), and with groups (80%)	–82% of the training schemes provided psychodynamic psychotherapy, 68% CBT, 46% integrative psychotherapy, and 46% systemic psychotherapy –Personal psychotherapy was mandatory in 53% of the centers –48% of the centers paid the full (27%) or partial (22%) costs for PT –In the Netherlands, PT was paid for by all centers (mostly full coverage) –46% PT was publicly funded (35% full, 11% partial coverage)
Kuzman et al. [28]	Croatia	Psychiatric trainees ***n* = 66** (RR: 89%)	–57% showed a high interest in psychotherapy	–65% were engaged in various PT activities –The majority of participants engaged privately in PT –Participants engaged in psychotherapy had a higher affinity for psychotherapy and a lower interest in pharmacotherapy	–18% in group analytic psychotherapy, 18% in CBT, 18% in psychoanalytic psychotherapy, and 18% in family psychotherapy
McNeill and Ingram [29]	UK Northern Ireland	Psychiatric trainees ***n* = 51** (RR: 52%)	–91% were aware of the requirements	–68% attended theoretical lectures on PT –The standard of theoretical lectures was rated as above average, 70% had problems getting access –35% had some experience in both CBT and psychodynamic psychotherapy –94% did not meet the mandatory requirements defined in the guidelines for PT	–CBT (56%) and psychodynamic psychotherapy (53%) were the two main types of training –28% had some experience in other modalities (i.e., group and systemic therapy)
Oakley and Malik [30]	Europe	Psychiatric trainee representatives ***n* = 22** (RR: 100%)	-	–PT was varied and compulsory in only 12 countries –In three countries, personal therapy was compulsory	–Access to PT: psychodynamic psychotherapy (91%), CBT (95%), family therapy (77%), systemic therapy (72.7%), interpersonal therapy (54.5%), DBT (31.8%)
Fiorillo et al. [31]	Europe	ECP representatives ***n* = 12** (RR: 92%)	-	–PT was mandatory in 10 countries, mostly with additional fees –More than half of the countries did not have clear guidance regarding trainees’ PT evaluation –25% used logbook or workplace-based assessment for evaluation, 33% used written or oral examination –In 75% of the countries, personal psychotherapy was mandatory; however, most trainees had to pay for it	–Training in psychodynamic therapy and CBT was available in almost all countries –Training in systemic therapy was provided in 50% of the countries, interpersonal, supportive, and psychoeducational techniques were available in 33%, and DBT in 25%
Simmons et al. [32]	Europe	Psychiatric trainee associations with CAP schemes ***n* = 28** (RR: 100%)	-	–PT in any form was obligatory in 68% of the countries –In 82% of the countries, PT was both theoretical and practical; in 14% of the countries, trainees had access to only theoretical PT –In 4%, there was no psychotherapy experience available –17.8% of the countries required personal therapy during PT, funded by training schemes in Finland and parts of Germany, and a contribution was paid in the Netherlands	–Psychodynamic training (67%) and CBT (78%) were the most common, followed by systemic therapy (64%)
Van Effenterre et al. [33]	France	Psychiatric trainees ***n* = 27**	–Trainees viewed psychotherapy as an integral part of their identities as psychiatrists	-	–Psychoanalysis was not in a hegemonic position anymore, and trainees’ demands were accordingly
Pinto da Costa et al. [34]	Portugal	Psychiatric trainees ***n* = 80** (RR: 41.5%)	–87.2% reported that PT should be included in the obligatory training	–31.6% had a formation in psychotherapy –Trainees in their last years were attending PT in higher percentages (57.7–18.9%) –Trainees from general hospitals (40.7%) were more likely to have PT compared to trainees from psychiatric hospitals (12%)	–Trainees had formation in CBT (60%), psychodrama (28%), interpersonal (20%), family (16%), and psychodynamic (8%) therapies –PT elected to be included as obligatory were CBT (62.5%), family (42.5%), and psychodynamic (26.3%) therapies
Van Effenterre et al. [14]	France	Psychiatric trainees ***n* = 869** (RR: 65%)	–The vast majority were interested in psychotherapy	–95% preferred a two-phase modality of training (general theoretical training and in-depth training in the preferred modality); however, the current training was different	-
Ertekin et al. [35]	Turkey	Psychiatric trainees (*n* = 107) ECPs (*n* = 33) ***n* = 140**	–78.5% were interested in psychotherapy	–32.1% had training in CBT during psychiatry training –24.3% attended PT on CBT outside of their training institution –76.4% used psychotherapy in their practice	–40.7% reported CBT as the type of psychotherapy they were most interested in, followed by psychoanalytic psychotherapy (35.5%)
Schmidt and Foli-Andersen [36]	Denmark	Psychiatric trainees ***n* = 60** (RR: 100%)	–Almost all were interested in psychotherapy –88% thought PT was relevant to psychiatry training and 66% thought it should meet UEMS recommendations	–PT was mandatory to qualify as a psychiatrist (conduct 60 sessions, 45 h of theoretical training, 45 h of supervision: 150 h total) –73% received no training beyond the mandatory courses in psychotherapy; the reasons were lack of time (86%), financial constraints (38%), and lack of interest (17%)	–PT was divided equally between CBT and psychodynamic psychotherapy –36% and 53%, respectively, had difficulties securing psychodynamic and CBT supervision
Levi Shachar et al. [37]	Israel	Psychiatric trainees (*n* = 72) Psychiatrists (*n* = 157) ***n* = 229** (RR: 15.3%)	–95.7% had positive attitudes toward psychotherapy and showed a willingness to use it –97.4% thought PT should be included in psychiatry training	–58.8% thought psychotherapeutic treatments were not available in their workplace, and 56.1% reported that professional knowledge in psychotherapy (i.e., professional journals, seminars, staff meetings, and journal clubs) was less than adequate –65.3% of trainees and 77.7% of specialists received personal psychotherapy, and those who had more favorable attitudes, higher levels of knowledge, and skills related to psychotherapy	–84.7% of trainees and 99.3% of specialists delivered psychotherapy during psychiatry training (mainly psychodynamic and CBT) –76.1% of the junior trainees delivered psychotherapy, and 100% of the senior trainees delivered psychotherapy during their training
Fàbrega Ribera and Ilzarbe [38]	Spain	Psychiatric trainees ECPs ***n* = 55** (RR: 59%)	–Participants were interested in in–depth training in psychotherapy	–46% received PT during their training	–46% received specific PT, mainly CBT (27.3%), third generation such as motivational interviewing or DBT (27.3%), psychodynamic (12.7%), and systemic (10.9%) therapies
Gargot et al. [12]	Europe	Psychiatric trainees ECPs ***n* = 572** (RR: 2.9% of estimated 20,000 European ECPs)	–92% considered psychotherapy as important for their professional identity –90% wanted to practice psychotherapy after psychiatry training –The majority were interested and motivated, but available resources appear scarce –60% would give >5% of their salary for PT	–48% were involved in PT and 5% completed an entire PT –Except for Malta, Germany, and Israel, <50% of respondents received any PT within psychiatry training –One out of five trainees reported having received PT –During PT, theoretical lectures (70%), practice (43%), and personal psychotherapy (22%) were compulsory at different rates –Only 36% of theoretical lectures and 26% of supervision were funded publicly –40% underwent personal psychotherapy –38% qualified for psychotherapy at the end of psychiatry training –UEMS recommendations were known by 51% and implemented in 22% of respondents’ country of origin	–60% were interested in psychodynamic therapy, 46% in CBT, 26% in systemic therapy, and 1% in group therapy
Barrett et al. [39]	Europe	Trainees or ECP representatives of national societies of ESCAP ***n* = 33** (RR: 86.8%)	-	–Psychotherapy theoretical topics were taught in 90% of the countries during the CAP training –Among countries reporting data on training for any of the psychotherapies (84.8%), 42.8% required practical training in individual psychotherapy with children, and 25% recommended it	–Among countries reported training on systemic therapy (81.8%), 22% required this training, and 18.5% recommended it
Revet et al. [40]	France	Child and adolescent psychiatrists ***n* = 668** (RR: 77.4%)	–One of the main reasons for choosing to specialize in CAP was interest in psychotherapy (52.8%)	–Personal psychotherapy was one of the identified personal factors in choosing CAP (22.2%)	-
Kaya et al. [41]	Turkey	Psychiatric trainees ECPs ***n* = 103**	–89.3% considered that psychotherapy should be included in their training	–68% received PT included in the psychiatry training –89.3% conducted psychotherapy themselves and 76.7% received supervision –40.8% had both theoretical and practical training, 16.5% received only theoretical training, 11.7% received only practical training –41.7% had personal psychotherapy –40.8% were certified	–The most common modalities were CBT (73.8%), psychodynamic therapy (51.5%), interpersonal therapy (8.7%), family therapy (8.7%), psychodrama (5.8%), and others (9.8%, sex therapy, EMDR, and schema therapy)
Yazici et al. [42]	Turkey	Psychiatric trainees ***n* = 113**	–85.7% thought that psychotherapy was an effective method –90.3% planned to continue structured PT after completing psychiatry training –100% thought they would need structured PT outside their training institution	–80.5% received theoretical PT, 63.7% at their institution –62.6% received supervision, 63.7% at their institution, 89% from other centers –51.8% applied structured psychotherapy, 11.5% felt fully competent –29.8% had 200–400 h of theoretical PT, 29.8% had 50–100 h, and 22.8% had <50 h (overall) –For 47.8%, PT was provided by faculty members of the institution	–The most common modalities were CBT (67.3%), supportive therapy (22.1%), sex therapy (17.7%), psychodynamic psychotherapy (15.9%), acceptance and commitment therapy (13.3%), metacognitive therapy (7.1%), and schema therapy (7.1%) –99.1% thought CBT and 65.5% supportive therapy should be compulsory in a medical training center
Region of the Americas					
Khurshid et al. [43]	USA	Chief psychiatric trainees ***n* = 72** (RR: 70.6%)	–84% were aware of competency requirements –54% viewed competency determination as having a positive impact –For 32%, competencies were well integrated into their training curriculum	–Psychotherapy competency was mainly determined by global assessment by nonphysician supervisors in 61% of programs –Case formulations (65%), specified reading materials (55%), and videos (46%) were used as instruments for PT –Majority reported a requirement of one to four patients for competency	-
Hadjipavlou and Ogrodniczuk [44]	Canada	Psychiatric trainees ***n* = 385** (RR: 63%)	–99% believed that psychotherapy has a vital role in psychiatric practice –For the majority, the ability to practice psychotherapy was an important aspect of their psychiatric identities –For 68%, learning psychotherapy was a factor in becoming a psychiatrist	–72% thought that PT was sufficiently emphasized in their training, and 75% felt that they were provided with broad training in different psychotherapy modalities –77% believed that their competence in different psychotherapy modalities should be formally assessed –84% anticipated practicing psychotherapy in the future –The percentage of trainees who planned to practice psychotherapy decreased with training –Trainees satisfied with their PT were more likely to anticipate practicing psychotherapy after graduation	–74% identified their psychotherapeutic orientations as eclectic –Among fifth-year trainees, 87% received training in long-term therapy, 58% in interpersonal psychotherapy, 93% in CBT, 56% in brief dynamic therapy, and 70% in group therapy
Petersen et al. [45]	USA	Psychiatrists ***n* = 66** (RR: 49%)	–All cohorts expressed a significant preference for psychopharmacology training over training in behavioral therapies	–Regarding specific job activities, psychodynamic therapy was present for 44% and supportive therapy for 38% across all three cohorts	–CBT was among the inadequately covered topics during training for all –Psychodynamic therapy and CBT training were ranked relatively high by trainees as being emphasized in psychiatry training –There was a significant preference for CBT training over psychoanalysis, but not over psychodynamic therapy training
Katz and Kaplan [46]	USA	Psychiatric trainees ***n* = 75** (RR: 42%)	–The importance of psychotherapy was seen as equal to biological treatments –76% rated the likelihood that they would practice psychotherapy	–General psychotherapy skills were rated adequate, good, or excellent by 65% –The majority did not believe they would pursue psychoanalytic training; the most common reasons were financial reasons and time commitment	–46% expressed interest in further psychodynamic PT, 22% in psychoanalytic training –56% reported limited knowledge of psychoanalysis
Khawaja et al. [47]	USA	Chief psychiatric trainees ***n* = 18** (RR: 100%)	–The majority of the trainees had low to moderate interest in learning group psychotherapy	-	–Formal group therapy training was absent in the first and second years of training and was optional in the third and fourth years –Six out of 18 programs exposed trainees to inpatient group therapy
Lanouette et al. [48]	USA	Psychiatric trainees ***n* = 249** (RR: 43.9%)	–81% viewed becoming a psychotherapist as integral to their psychiatric identities –79% were proud to be a psychotherapist –40% agreed that conducting psychotherapy was the most rewarding aspect of their work	–54% planned to provide formal psychotherapy	-
Zisook et al. [49]	USA	Psychiatric trainees ***n* = 249** (RR: 43.9%)	–11.8% reported decreased interest in psychotherapy during training; this rate was 16.4% for last year’s trainees –Decreased interest in psychotherapy correlated with personal and institutional factors	-	–Lower self-perceived competence in CBT and psychodynamic psychotherapy correlated with decreased interest in psychotherapy
Kovach et al. [50]	USA	Psychiatric trainees ***n* = 133** (RR: 40.5%)	-	–Current PT for trainees was highly variable –Trainees preferred more hours of didactic instruction in PT –Ranked teaching modalities were: Supervision>hours of psychotherapy performed>didactic instruction>readings>personal psychotherapy	–More training in interpersonal psychotherapy, DBT, couples, family, group, and child psychotherapy was needed
Radu et al. [51]	Canada	Psychiatric trainees ***n* = 18** (RR: 72%)	–67% considered highly or moderately being a psychotherapist as part of their identity –Conducting psychotherapy was highly rewarding (50%), psychotherapy skills were considered highly necessary to be a competent psychiatrist (83%), and 94% planned to incorporate psychotherapy into future practice to some extent	-	-
Morrissette et al. [52]	Canada	Psychiatrists ***n* = 21** (RR: 35.6%)	–90.5% reported that psychotherapy skills were very useful for nonformal psychotherapeutic aspects of their psychiatric practice –47.6% were very confident in their ability to help patients with psychotherapeutic treatments	–76.2% indicated that less than one-third of their current practice was dedicated to formal psychotherapy –76.2% felt that significant elements of psychotherapy knowledge or skills were learned after completing training	–Changes they would implement were opportunities for trainees to observe others providing psychotherapy and exposure to various modalities of psychotherapy
Ng et al. [53]	Canada	Psychiatric trainees ***n* = 146** (RR: 77%)	–94% planned to offer some form of psychotherapy for MDD and reported sufficient didactic teaching (86%) and supervision (81%) in the use of these modalities	–47% reported self-assessed competence in delivering multiple modalities –76% expressed interest in practicing multiple psychotherapeutic modalities	–Most reported receiving adequate training in CBT (83%) and behavioral activation (61%), followed by interpersonal psychotherapy (39%) and mindfulness-based cognitive therapy (22%) –59% reported competence in CBT
Belinati Loureiro et al. [54]	Brazil	Psychiatric trainees (*n* = 93) ECPs (*n* = 38) ***n* = 131**	–97.7% indicated that psychotherapy should be included in the training of psychiatrists –73.3% believed PT should be mandatory	–PT was included in psychiatry training (85.5%), primarily mandatory (78.6%) –One-third (30.5%) only theoretical, one-third (38.2%) both theoretical and practical training –57.3% reported receiving some form of PT during psychiatry training –26.7% had not received any PT	–Mostly CBT (65.6%), followed by psychodynamic psychotherapy (60.4%), interpersonal psychotherapy (13.5%), psychoanalysis (10.4%), psychodrama (8.3%), family therapy (7.2%), EMDR (3.1%)
South-East Asian region					
Kishore et al. [55]	India	Psychiatrists ***n* = 84** (RR: 37%)	–Psychiatrists felt that most of their patients needed psychotherapy (88%) –PT rates and psychotherapy practice rates were not associated	–64.3% of the participants received training in psychotherapy, and 35.7% received none –85.7% used psychotherapy as a treatment modality in their daily practice –Among those who had no formal PT, 85% practiced psychotherapy	–Most employed more than one method of therapy: Cognitive therapy (47.6%), behavior therapy (52.4%), psychodynamic psychotherapy (26.2%), and family therapy (52.4%) were the techniques commonly used
Grover et al. [56]	India	ECPs ***n* = 451** (RR: 22.7%)	-	-	–Available psychotherapy training modalities were behavior therapy (90.3%), supportive psychotherapy (87.6%), CBT (86.5%), and motivational interviewing (85.7%) –Most common psychotherapy modalities the participants carried out during training were behavior therapy (87%), supportive psychotherapy (83.2%), motivational interviewing (81.4%), and CBT (77.3%)
Rai et al. [57]	Nepal	Psychiatric trainees ECPs ***n* = 51** (RR: 53.7%)	-	–Mostly (72.5%) PT was included in psychiatry training, mostly mandatory (67.6%), and only theoretical (78.4%) –One-third (35.3%) had PT experience	–Mostly CBT (94.4%) Interpersonal psychotherapy (50%) Family therapy (38.9%) Psychodynamic psychotherapy (22.2%) Group therapy (5.6%) Motivational enhancement therapy (5.6%)
Kumar et al. [58]	India	Psychiatrists ***n* = 367** (RR: 29.3%)	-	–PT was included in the curriculum (73.8%), mainly as didactic lectures (74.1%) –53.7% were allowed to conduct psychotherapy sessions under supervision, while 70.8% conducted independent sessions –Half (53.1%) had no separate clinical rotation of psychotherapy, (52.6%) no forum, or psychotherapy meeting to evaluate and provide feedback on PT, (51.8%) no record or logbook, and (59.4%) no policies/guidelines related to PT –46.9% had separate PT during postgraduation –74.1% had lectures on psychotherapy in their institute/department during postgraduation	-
Western Pacific region					
Foulkes [59]	Australia and New Zealand	Senior psychiatric trainees ***n* = 94** (RR: 35%)	–PT was perceived as very important in the training of a psychiatrist, and there was a marked deficiency in the quantity of time devoted to PT	–Marked variations in the number of seminars and supervision within the same institution were indicated	–Most PT was received in dynamic and supportive therapy, followed by CBT, family therapy, and group therapy –Supportive therapy was considered the most commonly applicable therapy for patients seen, yet underrated in training
Lee et al. [60]	Republic of Korea	Senior psychiatric trainees ***n* = 65** (RR: 51.5%)	–A gradually increasing trend as the year of training increased was observed regarding the start of PT during psychiatry training –One quarter started PT in their second year, and one quarter in their third year	–Each trainee treated five psychotherapy cases during psychiatry training on average –Average frequency of psychotherapy sessions was once a week –61.3% conducted psychotherapy during regular working hours –When conducting psychotherapy, 59.2% recorded the session, 47.5% memorized the therapy	–67.4% practiced insight-oriented psychodynamic psychotherapy, 28.5% practiced supportive psychotherapy, and 4.2% practiced psychoanalysis (study excluded CBT)
Tor et al. [61]	Singapore	ECPs Psychiatric trainees ***n* = 54** (RR: 49.1%)	–Participants rated psychotherapy treatment knowledge during training as important	-	-
Charernboon and Phanasathit [62]	Thailand	ECPs ***n* = 82** (RR: 57.7%)	–98.8% believed that psychotherapy played an important role in being a psychiatrist –80.5% were willing to perform psychotherapy –41.5% lacked self-confidence in practicing psychotherapy	–PT was highly variable between institutes –67.1% indicated that the PT they received was adequate –86.6% needed further PT, mostly in CBT (42.7%), Buddhist psychotherapy (32.9%), and family therapy (28%)	–Most common modalities practiced were supportive psychotherapy (82.9%), CBT (39%), behavioral therapy (35.4%), Satir’s systemic psychotherapy (32.9%), family therapy (28%), systemic psychotherapy (32.9%), and Buddhist psychotherapy (28%)
Jacobson et al. [63]	Australia and New Zealand	Psychiatric trainees ***n* = 203** (RR: 21%)	–Over 80% regarded psychotherapy as important or very important –For 60%, it was more important than training in any psychiatric subspecialty	-	–The highest-rated modalities in terms of significance were CBT, supportive psychotherapy, and psychodynamic psychotherapy
Hongsanguansri et al. [64]	Thailand	Child and adolescent psychiatrists – graduated within the past 10 years ***n* = 60** (RR: 54%)	–Psychotherapy was rarely prescribed as a psychosocial intervention	–PT significantly varied across institutions –Psychodynamic psychotherapy, group psychotherapy, and family therapy, which were required core competencies of psychotherapy in CAP training, were not routinely used in everyday practice	–76.3% were trained in Satir’s transformational systemic therapy, 72.9% in CBT, and 72.9% in supportive psychotherapy –Behavioral therapy, supportive psychotherapy, and CBT were the most common modalities used

Abbreviations: CAP, child and adolescent psychiatry; CBT, cognitive behavioral therapy; DBT, dialectical behavior therapy; ECP, early career psychiatrist; EMDR, eye movement desensitization reprocessing; ESCAP, European Society for Child and Adolescent Psychiatry; MDD: major depressive disorder; PT, psychotherapy training; RR, response rate; UEMS, Union Européenne de Médecins Spécialistes-European Union of Medical Specialists.

**Figure 2.**
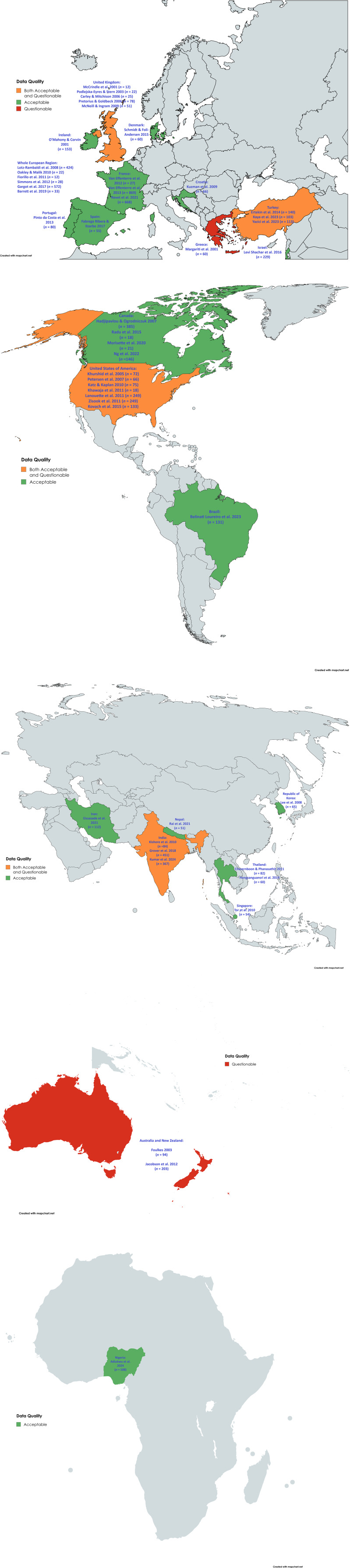
Quality assessment of included studies – World Map Charts.

Among the included studies published between January 1, 2000, and August 1, 2024, 47 reported quantitative results, 3 incorporated qualitative components following the survey [40, 46, 59], and 1 had primarily qualitative methodology [33]. According to the quality assessment of the 47 included studies using Q-SSP, 38 (80.9%) articles demonstrated acceptable quality, while 9 (19.1%) articles had questionable quality (Table 2). Considering a 60% threshold resulting in better consensus between experts [18], 40 (85.1%) were identified as having acceptable quality. One study with qualitative methodology showed reasonable quality according to the Evaluation Tool for Qualitative Studies. World maps created with mapchart.net demonstrate the results of the quality assessment of each included study (Figure 2).

**Table 2.** Quality assessment of included studies with Q-SSP

Study	Introduction (rational, questions, variables)	Participants (sampling, recruitment)	(Collection, analyses, measures, results, and discussion)	Ethics	Overall score	%	Result
Margariti et al. [21]	3/4	2/3	5/9	0/1	10/17	58.8	Q
McCrindle et al. [22]	4/4	3/3	5/9	0/1	12/17	70.5	A
O’Mahony and Corvin [23]	2/4	3/3	7/9	1/1	13/17	76.4	A
Foulkes [59]	3/4	2/3	5/9	0/1	10/17	58.8	Q
Podlejska-Eyers and Stern [24]	4/4	3/3	6/10	0/1	13/18	72.2	A
Khurshid et al. [43]	4/4	3/3	5/9	1/2	13/18	72.2	A
Carley and Mitchison [25]	4/4	3/3	4/9	1/1	12/17	70.5	A
Pretorius and Goldbeck [26]	4/4	3/3	6/9	1/1	14/17	82.3	A
Hadjipavlou and Ogrodniczuk [44]	4/4	3/3	8/9	2/2	17/18	94.4	A
Petersen et al. [45]	4/4	3/3	8/10	0/1	15/18	83.3	A
Lee et al. [60]	4/4	3/3	7/9	0/1	14/17	82.3	A
Lotz-Rambaldi et al. [27]	4/4	3/3	5/9	0/1	12/17	70.5	A
Kuzman et al. [28]	4/4	3/3	6/9	0/1	13/17	76.4	A
McNeill and Ingram [29]	3/4	3/3	4/9	1/1	11/17	64.7	Q
Katz and Kaplan [46]	4/4	3/3	8/9	1/2	16/18	88.8	A
Kishore et al. [55]	4/4	2/3	4/9	1/1	11/17	64.7	Q
Oakley and Malik [30]	3/4	3/3	4/9	0/1	10/17	58.8	Q
Tor et al. [61]	4/4	3/3	7/9	2/2	16/18	88.8	A
Charernboon and Phanasathit [62]	4/4	3/3	8/9	2/2	17/18	94.4	A
Fiorillo et al. [31]	4/4	3/3	3/9	0/1	10/17	58.8	Q
Khawaja et al. [47]	3/4	2/3	2/9	0/1	7/17	41.1	Q
Lanouette et al. [48]	4/4	3/3	8/9	2/2	17/18	94.4	A
Zisook et al. [49]	4/4	3/3	8/9	1/2	16/18	88.8	A
Jacobson et al. [63]	1/4	3/3	4/9	0/1	8/17	47.0	Q
Simmons et al. [32]	4/4	3/3	5/9	1/1	13/17	76.4	A
Pinto da Costa et al. [34]	4/4	3/3	7/9	1/1	14/17	88.2	A
Van Effenterre et al. [14]	4/4	3/3	5/9	0/1	12/17	70.5	A
Ertekin et al. [35]	4/4	1/3	3/9	0/1	8/17	47.0	Q
Kovach et al. [50]	4/4	3/3	8/9	2/2	17/18	94.4	A
Radu et al. [51]	4/4	3/3	7/9	1/2	15/18	83.3	A
Schmidt and Foli-Andersen [36]	4/4	3/3	6/10	2/2	15/19	78.9	A
Levi-Shachar et al. [37]	4/4	3/3	7/8	0/1	14/16	87.5	A
Fàbrega Ribera and Ilzarbe [38]	4/4	3/3	7/9	1/1	15/17	88.2	A
Gargot et al. [12]	4/4	3/3	8/9	1/1	16/17	94.1	A
Grover et al. [56]	4/4	3/3	9/10	2/2	18/19	94.7	A
Barrett et al. [39]	4/4	3/3	7/9	1/1	15/17	88.2	A
Hongsanguansri et al. [64]	4/4	3/3	7/9	1/1	15/17	88.2	A
Morrissette et al. [52]	4/4	3/3	6/9	2/2	15/18	83.3	A
Eissazade et al. [13]	4/4	3/3	7/9	1/1	15/17	88.2	A
Rai et al. [57]	4/4	3/3	8/9	1/1	16/17	94.1	A
Revet et al. [40]	4/4	3/3	8/9	1/1	16/17	94.1	A
Ng et al. [53]	4/4	3/3	8/9	2/2	17/18	94.4	A
Belinati Loureiro et al. [54]	4/4	3/3	7/9	1/2	16/18	88.8	A
Kaya et al. [41]	4/4	3/3	6/9	2/2	15/18	83.3	A
Yazici et al. [42]	4/4	3/3	7/9	2/2	16/18	88.8	A
Adiukwu et al. [20]	4/4	3/3	8/9	1/1	16/17	94.1	A
Kumar et al. [58]	4/4	3/3	8/9	2/2	17/18	94.4	A

Abbreviations: A, acceptable quality; Q, questionable quality; Q-SSP, quality assessment checklist for surveys in psychology.

Three-quarters of the studies reported data across the European region (*n* = 24, 50%) and the region of the Americas (*n* = 12, 25%). The remaining ones were collected from the Western Pacific region (*n* = 6, 12.5%), the South-East Asian region (*n* = 4, 8.3%), the Eastern Mediterranean region (*n* = 1, 2%), and the African region (*n* = 1, 2%). All studies evaluated psychiatry trainees’ and/or ECPs’ experiences with psychotherapy training by applying surveys, questionnaires, or semi-structured interviews. The majority of the studies used different, unvalidated questionnaires, mostly developed by the authors. Five (10.4%) studies applied uniform questionnaires titled the World Psychotherapy Survey as part of broader international research [13, 20, 41, 54, 57]. The number of respondents for each article varied from 12 to 869. Reported response rates were highly variable, ranging from 2.9 to 100%. A few studies mentioned attrition rates of 5% [36], 4.9% [56], and 4.6% [45]. A total of 7,196 participants were represented across studies, of whom 3,780 and 1,456 were indicated as psychiatric trainees and ECPs, respectively. Seven studies were conducted with only senior trainees or specialist registrars, pointing to an advanced level of training or trainees in their last year of training [22, 26, 38, 43, 47, 59, 60]. In five studies, representatives of trainee or ECP associations were surveyed regarding the system in their countries of training [27, 30–32, 39]. Child and adolescent psychiatry (CAP) trainees and/or ECPs were included in four studies focusing on psychotherapy training in CAP. Among those, 728 ECPs and 61 representatives of trainee associations were surveyed [32, 39, 40, 64]. One study reported the views of CAP trainees and ECPs as part of the whole group [41]. In the following sections of the article, the term ECP refers to both psychiatry trainees and ECPs, if not otherwise indicated.

Thirty-one of the included studies reported data regarding ECPs’ interest in psychotherapy training. Overall, 57–80% (minimum–maximum) of the participants were interested in psychotherapy training [14, 27, 28, 35, 36], and 75.8–99% mentioned the importance of such training in becoming fully competent psychiatrists [12, 23, 42, 44, 51, 55, 59, 62, 63, 52]. ECPs demonstrated a favorable/positive attitude toward psychotherapy training [36, 48], and 76–94% were willing to practice or advance it further [12, 42, 44, 46, 48, 51, 53, 62]. Moreover, 67–92% of the participants tended to consider being a psychotherapist as an integral and valuable part of their identities as psychiatrists [12, 44, 48, 51]. A considerable proportion of trainees wished for greater exposure to psychotherapy training (54%) and desired to develop a special interest (65%) [24, 26]. Psychotherapy interest was identified as one of the main factors in choosing to specialize in psychiatry [40, 44]. ECPs supported that psychotherapy training should be included in psychiatry training (88–97.7%) and it should be an obligatory part of the curriculum (57.2–87.2%) [13, 34, 36, 37, 41, 48, 54]. Among numerous psychotherapy modalities, CBT (62.5–100%), psychodynamic psychotherapy (26.3–71.4%), family/systemic therapy (42.5–71.4%), supportive psychotherapy (65.5%), and interpersonal psychotherapy (34.8–44%) were favored to be included in the curriculum to a variable extent [13, 34, 42, 54, 63]. Studies investigating the awareness of ECPs of psychotherapy training guidelines revealed that 51–84% had notice of competence requirements [12, 25, 36, 43]. In all, 33–48.8% reported being accredited or meeting the relevant requirements during the evaluation [26, 41]. Participants’ self-perceived psychotherapy competence rates were 47–65% [46, 52, 53].

Van Effenterre et al. found that psychiatry trainees viewed psychotherapy as central to their psychiatric identity and saw it as ranging from a strong therapeutic alliance to formal interventions. The psychotherapeutic orientation was diverse, with various modalities enriching psychiatry practice. Trainees expressed a desire for advanced theoretical and practical knowledge to maintain effective psychotherapy skills [33].

Psychotherapy training rates and opportunities in psychiatry training varied widely across countries, regions, institutions, and even within the same institution. Half of the studies were from Europe, where most countries considered psychotherapy training essential, although its inclusion in programs varied. It was mandatory in Denmark, Germany, Ireland, the Netherlands, Norway, Switzerland, and the United Kingdom [23, 26, 27], while it was optional in Belgium, France, and Portugal [31, 34]. Austria, Israel, Spain, and Turkey recommended it as a core competence and included it in the training to some extent [27, 37, 41]. The most accessible psychotherapy training modalities were CBT and psychodynamic psychotherapy, followed by family/systemic, supportive, and interpersonal psychotherapy [22, 24, 27, 29, 30, 32, 38, 41, 42]. In studies reporting data from Europe, CBT training was available for 18–95% of the participants [12, 22, 24, 27–30, 32, 34, 35, 38, 41, 42]. Psychodynamic psychotherapy training rates were 8–91% [12, 21, 22, 24, 25, 27, 28, 30–32, 34, 38, 39, 41], whereas 8.7–81.8% of the samples reported receiving training in systemic/family therapy [12, 22, 24, 25, 27, 28, 30–32, 34, 38, 39, 41]. Other modalities like interpersonal psychotherapy, supportive psychotherapy, group therapy, cognitive-analytical therapy, integrative/eclectic therapy, sex therapy, psychodrama, dialectical behavior therapy, and third-generation psychotherapies were offered to a more limited extent in European programs.

A quarter of the studies included were from the region of Americas. In Canada and the United States, psychotherapy training was a required competency [49, 52]. Ng et al. [53] reported that 83% of Canadian trainees received adequate CBT exposure, followed by 39% with interpersonal psychotherapy. In Brazil, psychotherapy training was mandatory in the core curriculum for psychiatry, with 78.6% of participants confirming its inclusion. The most accessible modalities were CBT, psychodynamic, and interpersonal psychotherapy [54].

Six studies from the Western Pacific Region provided data on psychotherapy training in Australia, New Zealand, the Republic of Korea, Singapore, and Thailand. In Australia and New Zealand, psychotherapy training varied by region and institution [59, 63], with psychodynamic psychotherapy, supportive psychotherapy, and CBT being the most accessible modalities [59]. In the Republic of Korea, psychotherapy was a requirement for certification, with 67.4% of the respondent trainees practicing insight-oriented psychodynamic psychotherapy, 28.5% supportive psychotherapy, and 4.2% psychoanalysis [60]. Singapore had high trainee interest but lacked standardized competency assessments [61]. In Thailand, psychotherapy training availability varied by institution, exacerbating challenges like heavy workloads, lack of confidence, and inadequate training [62]. Notably, Satir’s systemic therapy and Buddhist therapy were among the unique modalities practiced. The most common therapies were supportive psychotherapy (82.9%), CBT (39%), behavioral therapy (35.4%), and Satir’s systemic psychotherapy (32.9%).

Four studies from the South-East Asian region (three from India and one from Nepal) were included. In India, the National Medical Commission highlighted psychotherapy training as essential, but 64.3–73.8% of ECPs reported receiving it [55, 58]. The most common modalities included behavior therapy (90.3%), supportive psychotherapy (87.6%), CBT (86.5%), and motivational interviewing (85.7%) [56]. In Nepal, postgraduate psychiatry training lacked a uniform curriculum, and 67.6% of ECPs reported receiving mandatory psychotherapy training [57]. The most accessible modalities were CBT, interpersonal psychotherapy, and family therapy.

There was one study for each region that included the African and Eastern Mediterranean regions. Psychotherapy training was an integral rotation in psychiatry training in Iran [13]. In contrast, in Nigeria, it was not considered one of the mandatory rotations, with limited structured programs to incorporate psychotherapy into psychiatry training [20].

Four studies focused on psychotherapy training during CAP training programs. Revet et al. [40] mentioned that 52.8% of CAP trainees chose the specialization due to their interest in psychotherapy. Simmons et al. [32] reported that 19 of 28 European countries, namely Croatia, Czechia, Denmark, Estonia, Finland, Germany, Hungary, Ireland, Israel, the Netherlands, Norway, Poland, Portugal, Slovakia, Slovenia, Sweden, Switzerland, Turkey, and the United Kingdom, included mandatory psychotherapy training in CAP, with 82% offering both theoretical and practical experience. In Romania, there was no psychotherapy experience available at the time of that study. The most available modalities were CBT (78%), psychodynamic psychotherapy (67%), and systemic therapy (64%) [32]. The CAP-STATE study showed that 90% of countries taught theoretical psychotherapy topics, with 12 countries requiring practical training in individual psychotherapy with children. Systemic therapy training was provided by almost all countries (*n* = 27) [39]. In Thailand, psychotherapy training varied across institutions, with Satir’s transformational systemic therapy (76.3%), CBT (72.9%), and supportive psychotherapy (72.9%) being the most common modalities [64].

In studies reporting on psychotherapy training, theoretical training rates ranged from 41.6 to 97.6%, while practical training took place for 35.3–96% of participants [12, 13, 20, 22, 24, 25, 27, 29, 32, 39, 41, 42, 53, 54, 57, 58]. Psychotherapy training outside the psychiatry training institute occurred for 24.3–52% of trainees [12, 13, 22, 24, 25, 28, 32, 37, 39, 41, 54, 57, 58]. Van Effenterre et al. [14] stated that 95% of the psychiatry trainees preferred a two-phase training model, combining general theoretical training with in-depth training in a preferred modality, although current models were incompatible. Numerous training techniques, such as case formulations, reading materials, videos, logbooks, and workshops, were reported [31, 43]. Kovach et al. [50] noted that trainees ranked supervision highest among teaching modalities, followed by psychotherapy practice, didactic instruction, readings, and personal psychotherapy.

Funding for psychotherapy training was also a key issue. In Nigeria, 56.5% of ECPs self-funded their training [20]. In Europe, Lotz-Rambaldi et al. [27] reported that 48% of centers covered all (27%) or part (22%) of the costs, while 46% of training was publicly funded. Where not fully publicly funded, trainees often paid between 2,500 and 10,000 Euros, with some centers charging over 10,000 Euros (in Austria, Switzerland, and Germany) [27].

Interest in, accessibility to, and training modalities of psychotherapy training were compared between HICs and LMICs using results from 34 studies (Table 3). Others were excluded due to the lack of comparable country data and the fact that they were regional. Our results showed that, despite small discrepancies between countries, there were no significant differences in terms of interest in psychotherapy between HICs and LMICs.

**Table 3.** Psychotherapy training in high-income versus low- and middle-income countries

High-income countries					Low- and middle-income countries				
Country	Article	Interest in psychotherapy^a^	Access to psychotherapy training	Primary modalities	Country	Article	Interest in psychotherapy	Access to psychotherapy training	Primary modalities
Australia and New Zealand	Foulkes [59]	–	–	Psychodynamic Supportive CBT	Brazil	Belinati Loureiro et al. [54]	97.7%	57.3%	CBT Psychodynamic Interpersonal
	Jacobson et al. [63]	Over 80%	–	–	India	Kishore et al. [55]	88%	64.3%	CBT Family therapy Psychodynamic
Canada	Hadjipavlou and Ogrodniczuk [44]	84–99%	100%	–		Grover et al. [56]	–	–	Behavioral therapy Supportive CBT
	Radu et al. [51]	83–94%		–		Kumar et al. [58]	–	73.8%	–
	Morrissette et al. [52]	90.5%		–	Iran	Eissazade et al. [13]	–	98.2%	CBT Psychodynamic Family therapy/ Interpersonal
	Ng et al. [53]	94%		CBT Interpersonal Mindfulness-based	Nepal	Rai et al. [57]	–	72.5%	CBT Interpersonal Family therapy
Croatia	Kuzman et al. [28]	57%	65%	CBT Psychodynamic Family therapy	Nigeria	Adiukwu et al. [20]	–	52.8%	CBT Family therapy Psychodynamic
Denmark	Schmidt and Foli-Andersen [36]	Almost all of the sample	100%	CBT Psychodynamic	Thailand	Charernboon and Phanasathit [62]	98.8%	–	Supportive CBT Behavioral therapy
France	Van Effenterre et al. [14]	Vast majority	–	–		Hongsanguansri et al. [64]	–	–	Satir’s transformational systemic therapy CBT Supportive
Greece	Margariti et al. [21]	A considerable number	–	Psychoanalytic psychotherapy	Turkey	Ertekin et al. [35]	78.5%	–	–
Ireland	O’Mahony and Corvin [23]	75.8%	39.2%			Kaya et al. [41]	89.3%	68%	CBT Psychodynamic Interpersonal
Israel	Levi Shachar et al. [37]	95.7%	–	CBT Psychodynamic		Yazici et al. [42]	90.3%	80.5%	CBT Supportive Sex therapy
Republic of Korea	Lee et al. [60]	–	–	Psychodynamic Supportive (Study excluded CBT)					
Portugal	Pinto da Costa et al. [34]	87.2%	31.6%	CBT Psychodrama Interpersonal					
Spain	Fàbrega Ribera and Ilzarbe [38]	–	46%	CBT Third generation therapies Psychodynamic					
United Kingdom	McCrindle et al. [22]	–	83%	Psychodynamic CBT Family therapy					
	Podlejska-Eyers and Stern [24]	–	91%	Long-term psychotherapy CBT Family/marital therapy					
	Carley and Mitchison [25]	–	88%	Psychodynamic CBT Family therapy					
	Pretorius and Goldbeck [26]	80%	–	–					
	McNeill and Ingram [29]	–	68%	CBT Psychodynamic					
United States	Katz and Kaplan [46]	76%	100%	–					
	Lanouette et al. [48]	81%		–					

Abbreviation: CBT, cognitive behavioral therapy.

a Includes interest in psychotherapy in terms of mentioning the importance of the topic, demonstrating a favorable/positive attitude, willingness to practice, or advance further.

## Discussion

Our review of research from the past quarter century confirmed our hypothesis: psychiatry trainees and ECPs exhibit high interest in psychotherapy, which is consistent across different years and healthcare systems globally. However, opportunities for training were inadequate. Despite many ECPs advocating compulsory psychotherapy training in psychiatry, it was not consistently included as a required competency in curricula, except in a few HICs. Even where training was mandatory, significant variations in implementation across institutions and a lack of standardized education were common. Cognitive behavioral therapy and psychodynamic psychotherapies were the primary modalities offered in training.

Psychiatrists are uniquely positioned to integrate the biological and psychosocial aspects of healthcare, with psychotherapy knowledge, skills, and practices being essential to their professional identity. While psychopharmacological treatments have vastly advanced, current treatment algorithms recommend various evidence-based psychotherapy methods for common mental disorders, such as anxiety and depressive disorders [5]. Despite the diversity of these different approaches, there is a relatively similar efficacy effect size between psychotherapies. CBT, for instance, is as effective as medication in the short term and more effective in the longer term for depression [65]. Psychotherapy is also important for the treatment of severe mental disorders like schizophrenia [66].

### Psychotherapy in training guidelines

Psychotherapy training is integral to building therapeutic relationships, which are vital for treatment success, as outlined in UEMS, European Psychiatric Association, and WPA guidelines [8, 9, 67]. On the other hand, the UEMS and ACGME guidelines differ significantly in their psychotherapy training requirements. The ACGME mandates psychodynamic therapy, CBT, and supportive therapy for adult psychiatrists but does not require group therapy, systemic therapy, or family therapy [10]. For CAPs, CBT and family therapy are mandatory, while other therapies are recommended. In contrast, UEMS guidelines require all these therapies – psychodynamic therapy, CBT, systemic therapy, group therapy, supportive therapy, and family therapy – for both adult and CAPs [9]. This suggests that UEMS adopts a comprehensive approach, aiming to make therapists proficient in multiple treatment methods, whereas the ACGME provides more flexibility, allowing individual training programs to determine their psychotherapy training.

While no comparative studies directly assess the implementation of UEMS and ACGME criteria, certain challenges in applying both guidelines have been noted. In some European countries, psychotherapy training was still not mandatory, and the high costs associated with training were identified as a major barrier [27]. Expensive training may deter young psychiatrists from pursuing psychotherapy practice. To address this, psychotherapy training should be integrated into regular working hours, and training costs should be covered by the training institutions or relevant authorities. Ensuring a high standard of education and enhancing the effectiveness of UEMS at the national level are essential steps in improving psychotherapy training. Despite significant variability in the quality and availability of psychotherapy education, the integration of this training into psychiatry programs in many European countries is a notable achievement of the UEMS [27].

The applicability of the ACGME criteria indeed varied across institutions, highlighting a gap between theoretical guidelines and practical implementation [43]. While most trainees were aware of the qualification criteria, only about one-third believed these criteria were adequately incorporated into the specialty training curriculum. A study focused on psychiatric trainees’ expectations indicated a strong preference for more clinical psychotherapy experience, theoretical training, and supervision activities [50]. This underscores the need for further work in adapting these criteria to real-world training settings and ensuring that they are effectively implemented. Tailoring the criteria to the realities of psychiatry training programs could enhance the quality of psychotherapy education for trainees.

### Global challenges in psychotherapy training

Psychotherapy training faces similar barriers in both high-income HICs and LMICs, although the causes may differ [68]. In LMICs, challenges include limited resources, insufficient numbers of psychotherapy instructors, high training costs, and inadequate infrastructure. Psychiatry trainees reported inadequate supervision and discontinuity in training [64], and many lacked sufficient support due to financial and time limitations, as well as insufficient supervisory resources [42]. Cultural and systemic barriers exacerbate these challenges in LMICs. The absence of standardized psychotherapy curricula results in disparities in training quality, adversely affecting clinical practice. For instance, in Nigeria, psychotherapy training often remained insufficient and largely theoretical [20]. In India, many psychiatrists faced gaps in their educational needs, frequently having to complete psychotherapy training independently [55]. Socioeconomic factors also restrict the practice of psychotherapy after graduation; in Thailand, limited postgraduation supervision led to low confidence among psychiatry trainees, while cultural norms could further impede the acceptance of psychotherapy, reducing patient engagement [64].

In HICs, similar obstacles persist, including variability in training curricula, lack of supervised practice, and heavy clinical workloads [12, 14]. In Ireland, while a significant proportion of psychiatry trainees received formal psychotherapy training, it was primarily theoretical, with limited opportunities for supervised practice [23]. In the United States, psychiatry trainees valued psychotherapy training, but time and resource limitations prevented them from fully benefiting [43, 46]. In Canada, while trainees expressed satisfaction with the quality of supervision, they face difficulties developing therapeutic skills due to heavy clinical workloads and time constraints [44]. In addition, it was observed in the United States that psychiatry trainees’ interest in psychotherapy decreased as they progressed through their training, which was often attributed to institutional barriers, such as lack of supervision and high patient loads [49]. These findings are in line with decreasing empathy during medical school and residency, which might compromise striving for professionalism that eventually threatens the quality of health care [69].

Overall, regardless of the economic context, psychotherapy training is hampered by several systemic challenges, including a lack of supervision, intensive clinical schedules, time limitations, and insufficient funding. These factors hinder psychiatry trainees’ ability to effectively apply psychotherapy skills in their clinical practice postgraduation, thereby affecting their motivation and competence [27, 32].

### Interest in psychotherapy during training

A recently published review article reported that psychiatry trainees tended to lose interest in psychotherapy during the years of training. Dissatisfaction with the quality of psychotherapy curricula, a lack of support, and low self-perceived competence in psychotherapy were among the factors associated with a decreased interest in psychotherapy training [70]. In our review, two studies mentioned similar results, indicating that the percentage of trainees interested in psychotherapy or who planned to practice psychotherapy decreased with psychiatry training [44]. This rate was even higher among senior trainees [49]. Decreased interest in psychotherapy was correlated with personal factors like a lower endorsement of seeing psychotherapy as integral to one’s professional identity, not planning to provide a great deal of psychotherapy to patients, and not planning to pursue additional psychotherapy training. Institutional factors related to decreased interest in psychotherapy included negative attitudes toward the training program and dissatisfaction with the curriculum [49]. Trainees who felt satisfied with their psychotherapy training were more likely to anticipate practicing psychotherapy after graduation [44]. Moreover, lower self-perceived competence in CBT and psychodynamic psychotherapy correlated with decreased interest in psychotherapy.

### Practical recommendations to bridge the treatment gap

Despite the sizable evidence base and all the recommendations in treatment guidelines, there is a significant gap between the availability of effective psychotherapies and the delivery of such interventions in the community [71]. The WHO estimated that 76–85% of people with severe mental illnesses in LMICs and 35–50% in HICs receive no treatment for their conditions, and coverage of evidence-based mental health promotion and prevention approaches is even lower [72]. It can be argued that the burden of health systems on psychiatrists, economic, cultural, and political factors, as well as variations in psychotherapy training, might be prominent factors in this gap. In our study, we found that evidence-based psychotherapies ranked first among the prominent modalities in psychotherapy training worldwide. In addition, types of therapies integrated with each country’s own culture, such as Satir’s systemic therapy and Buddhist therapy in Thailand [62] or family therapy in Iran [13], were among the therapies that were of interest and at the forefront of the relevant training. From a broader perspective, it is recommended that CBT be applied in a culturally adapted way. In this context, one of the essential factors that should be considered for psychotherapy training in psychiatric education is the use of culturally adapted psychotherapy techniques and appropriate training methods.

Recent studies have reported that many psychotherapy methods are effective in the treatment of common mental illnesses and that there are no significant differences in effectiveness between evidence-based therapy modalities despite great differences in the quality of evaluation of this evidence [73]. To disseminate, develop, and sustain these practices, there is a need for psychotherapy training to continue its existence in institutions providing psychiatric education and to increase standardization by improving the training content. Considering that the need for training and supervision does not end after becoming a specialist, there is a need for programs that can ensure continuity. Perhaps an equally important task falls on professional organizations and associations that support continuous psychiatric education. Contributions of professional organizations in this context include organizing online or face-to-face seminars and workshops, journal clubs, case presentation sessions, regular psychotherapy training programs, or providing training materials, such as the online guidebook of the European Federation of Psychiatric Trainees [74], online courses of the European Psychiatric Association [75, 76], or the WPA’s online learnbook [77]. Keeping training and supervision focused on types of psychotherapy that are effective for mental health conditions is vital in terms of ensuring patients’ access to evidence-based treatment services and preventing potentially harmful practices [36]. Initiatives aiming to influence the learning outcomes of future psychiatric specialists, such as the European Board Examination in Psychiatry, which was held for the first time in 2025, could help achieve the goal of better and more accessible psychotherapy education for psychiatrists [78]. Future studies might evaluate the accessibility, adequacy, and effectiveness of such educational activities.

The limitations of our study include the cross-sectional study designs, medium-low response rates, and potential survey respondent bias, as self-reports may attract those more interested in psychotherapy. The variability in assessment tools and the evolving nature of psychiatric education over the last 25 years made it difficult to draw unified conclusions. In addition, the limited response rates and database selection may have reduced sample diversity, and the exclusion of non-English, French, or Turkish articles may have limited global applicability. Due to the heterogeneity of the data, a meta-analysis with sensitivity analysis was not included, which in turn may have limited the statistical robustness of the conclusions, even though most of the articles consistently reported high interest rates. Publication bias might have occurred in favor of promoting access to and training in psychotherapies, since these surveys could have been made by researchers most interested in psychotherapy and would be filled out by respondents with a similar profile. However, the studies were not funded by commercial interests. Preregistration, as was done by this review, can limit this bias. Beyond reported data, providing administrative teaching and training data would be useful to better understand real-life implementation [72].

Despite these limitations, to our knowledge, this is the first systematic review mapping psychotherapy training opportunities and highlighting possible areas for improvement. It is important to continue psychotherapy training in evidence-based modalities since they have the most evidence in terms of effectiveness across a wide spectrum of mental health conditions. It may also be useful to emphasize training for improving interpersonal skills and nonspecific factors independent of the psychotherapy modality. Further studies may focus on what can be done to increase feasibility and training in evidence-based therapies. Investigating the role of professional organizations and the effectiveness of online training could further enhance education in this field.

## Conclusion

Our review highlights a consistent interest in psychotherapy among psychiatry trainees and ECPs. However, despite this interest, the availability and quality of psychotherapy training remain highly variable across countries, regions of the countries, and institutions. This inconsistency poses a significant barrier to equipping future psychiatrists with the necessary skills to deliver evidence-based and culturally appropriate psychotherapeutic interventions.

Addressing this gap requires a coordinated effort. Formal training institutions, regulatory bodies that establish and monitor educational standards, and professional organizations responsible for continuing professional development all play critical roles. It is essential that these actors work together to ensure that psychotherapy training becomes an integral and standardized component of psychiatric education.

In an increasingly globalized world marked by growing mental health needs and complex societal challenges, it is essential to strengthen psychotherapy training. Doing so will not only enhance the competencies of future psychiatrists but also ensure better access to comprehensive mental health care for diverse populations.

## Supporting information

10.1192/j.eurpsy.2025.10044.sm001 Tanyeri Kayahan et al. supplementary material 1 Tanyeri Kayahan et al. supplementary material

10.1192/j.eurpsy.2025.10044.sm002 Tanyeri Kayahan et al. supplementary material 2 Tanyeri Kayahan et al. supplementary material

## Data Availability

The authors confirm that the data supporting the findings of this systematic review are available within the article and its Supplementary Materials.

## Supplementary material

The supplementary material for this article can be found at http://doi.org/10.1192/j.eurpsy.2025.10044.
