# Supplementary material for: Psychotherapy training in psychiatry: a systematic review on the early career psychiatrists’ interests and opportunities
Source: Eur Psychiatry. 2025 Jun 16;68(1):e88. doi: 10.1192/j.eurpsy.2025.10044 (PMC12260736; doi:10.1192/j.eurpsy.2025.10044)
Supplement: Tanyeri Kayahan et al. supplementary material 2 — Tanyeri Kayahan et al. supplementary material [file S0924933825100448sup002.docx]

**Supplementary Materials**

**Application of Quality Assessment Checklist for Survey Studies in Psychology (Q-SSP)**

Firstly, noting that most studies define objectives or aims rather than specific hypotheses or research questions, the former were accepted as eligible statements for item 3. For item 8, “response rate” was substituted for “attrition rate”. For item 9, to obtain a “yes” score, studies had to mention whether they used any strategies to minimize missing responses or nonresponse. For item 11 to obtain a “yes”, it was sufficient to state that a copy of all measures was available upon request. For item 12, it was considered sufficient to comment on the validity or lack thereof of the questionnaire used and/or to provide information about the validity of existing tools that the survey instrument included. For item 13, to obtain a “yes”, it was expected to comment on the professional characteristics of researchers who collected data, therefore, all studies received “no”. For item 16, to obtain a “yes”, it was sufficient for studies to include all the following key demographic characteristics: age, gender, and years or level of training/experience as a psychiatrist. For item 18 concerning informed consent, it was sufficient to report whether there was ethical review board approval or an exemption to obtain a “yes”. For item 19 regarding participant debriefing, as included studies did not involve participant deception, the “not applicable” code was used for all studies.
